# Supplementary figures and images for: Critical Dynamics in the Evolution of Stochastic Strategies for the Iterated Prisoner's Dilemma
Source: PLoS Comput Biol. 2010 Oct 7;6(10):e1000948. doi: 10.1371/journal.pcbi.1000948 (PMC2951343; doi:10.1371/journal.pcbi.1000948)

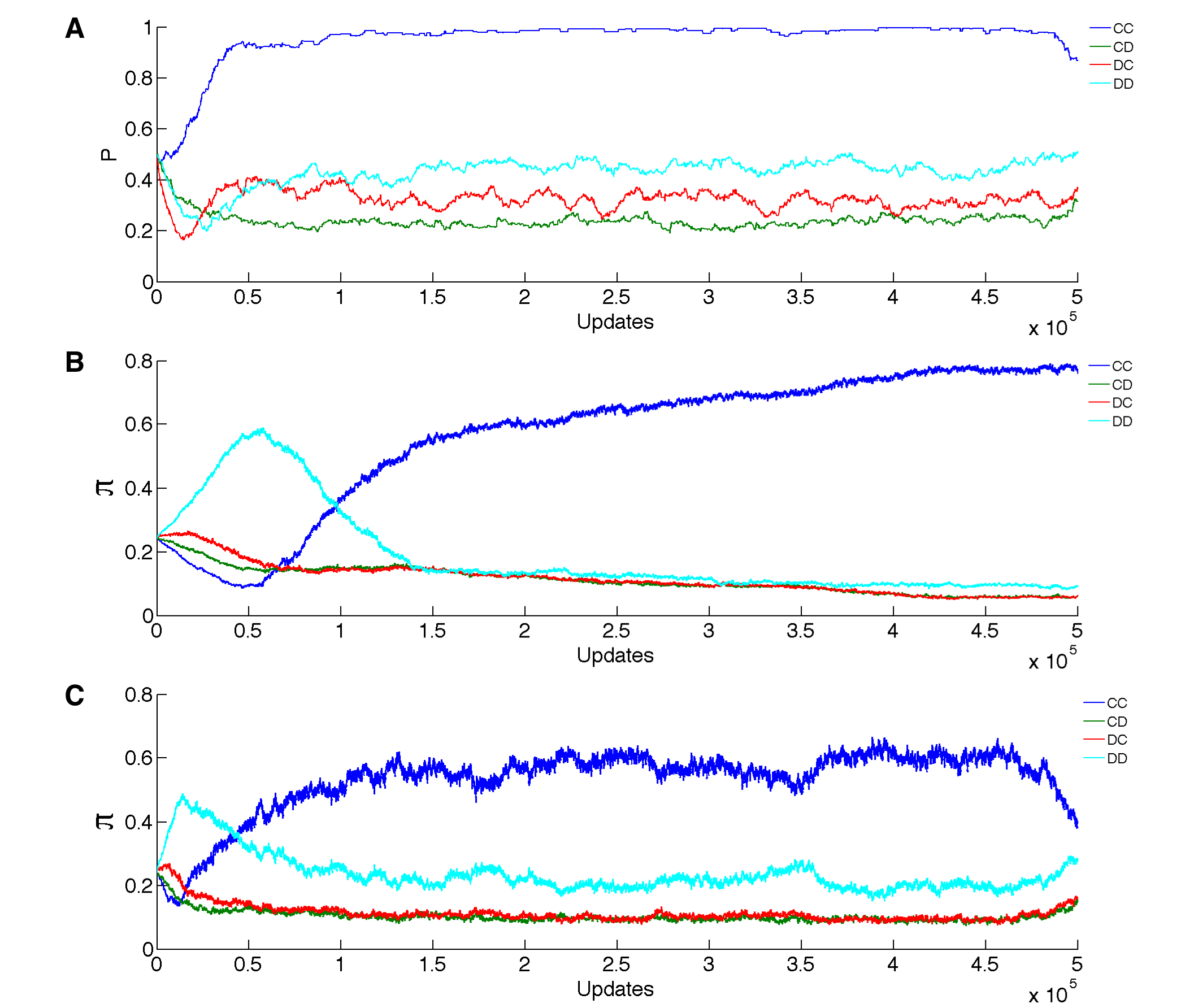

Supplement: Figure S1 — Average gene probabilities and play statistics. LOD gene probabilities (PXY) and play statistics (πXY) for a spatially-structured population, averaged over 80 experiments (500,000 updates each), at different μ and fixed r (1%). PC and πC are omitted because PC drifts almost neutrally (see Methods). (A) Average gene probabilities recorded at μ = 1%. (B) Play statistics recorded at μ = 0.1%. (C) Play statistics recorded at μ = 2%. (0.25 MB TIF) [file pcbi.1000948.s001.tif]

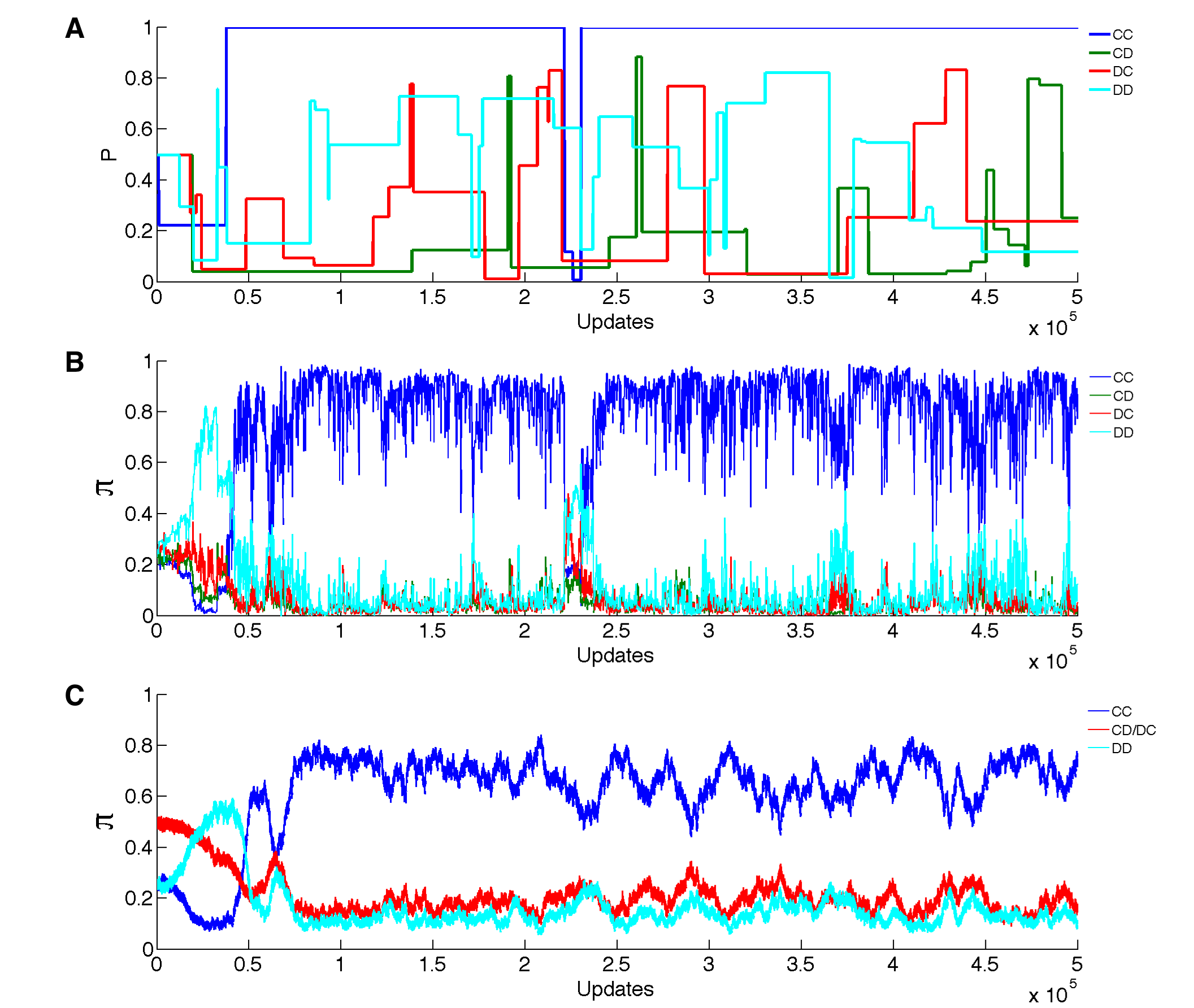

Supplement: Figure S2 — LOD and population genotypes and phenotypes. Single run LOD gene probabilities (PXY) and play statistics (πXY), as well as population average play statistics, for a spatially-structured population at μ = 1% and r = 1%. PC and πC are omitted because PC drifts neutrally (see Methods). (A) LOD gene probabilities. (B) LOD play statistics. (C) Average population play statistics. (0.41 MB TIF) [file pcbi.1000948.s002.tif]

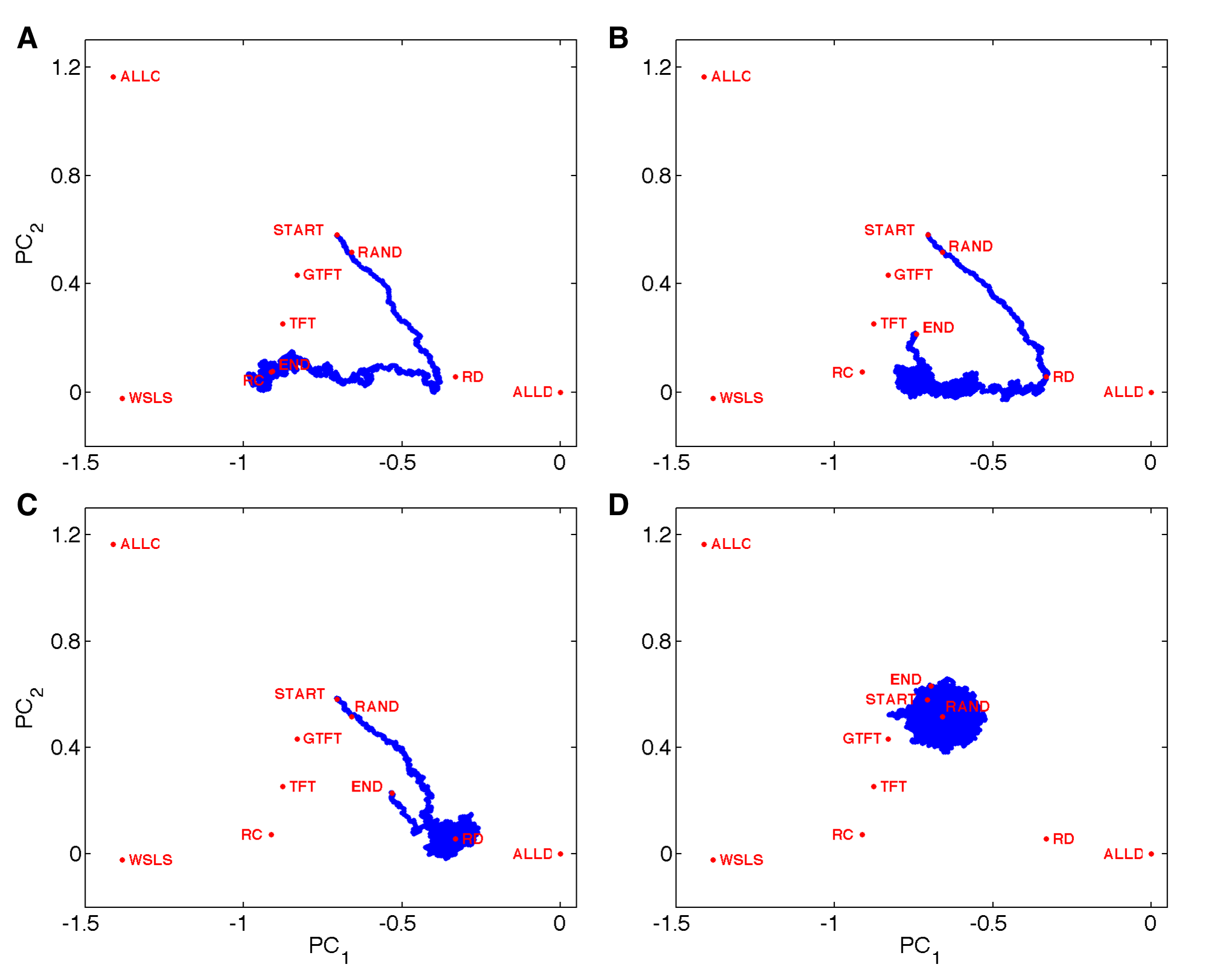

Supplement: Figure S3 — Evolutionary trajectories and attractors for well-mixed populations. All trajectories start at the same point (‘START’), and move towards the strategy marked by ‘END’. Several well-known strategies provide landmarks in strategy space: ‘TFT’: (PCC, PCD, PDC, PDD) = (1,0,1,0), ‘ALLC’ = (1,1,1,1), ‘ALLD’ = (0,0,0,0), WSLS = (1,0,0,1), GTFT = (1,0.333,1,0), START = (0.5,0.5,0.5). All experiments shown are run at replacement rate r = 1% for well-mixed populations. (A), Evolution of the average LOD for μ = 0.5%. RC marks the consensus genotype of this trajectory (described in supplementary text S1). This attractor is not the same as ‘END’ because that genotype lies past the most recent common ancestor of the population. (B) Trajectory for μ = 1.5%, close to the critical mutation rate. (C) Trajectory for μ = 5%. ‘RD’ marks the consensus genotype for these parameters. (D) Trajectory for μ = 50%. ‘RAND’ marks the consensus genotype for these parameters. (0.14 MB TIF) [file pcbi.1000948.s003.tif]

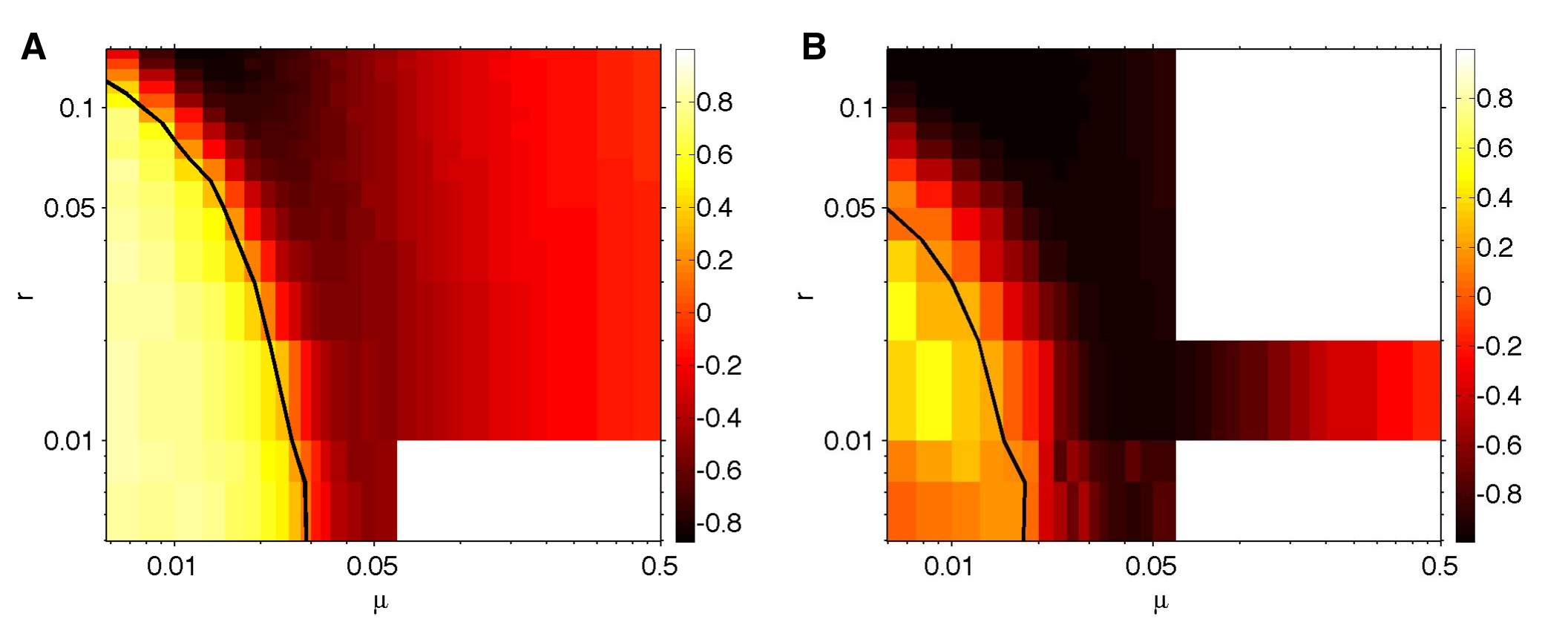

Supplement: Figure S4 — Quantitative phase transition diagrams as a function of μ and r. Coloring is applied according to the order parameter (m) with dark red to black indicating defection (m<−0.2), light yellow to light orange indicating cooperation (m>0.2) and orange indicating a transition regime of equal cooperation and defection (0.2≥m≥−0.2). White colored areas contain no recorded data. (A) Spatially-structured environment. (B) Well-mixed environment. (0.46 MB TIF) [file pcbi.1000948.s004.tif]
