# Supplementary material for: Critical Dynamics in the Evolution of Stochastic Strategies for the Iterated Prisoner's Dilemma
Source: PLoS Comput Biol. 2010 Oct 7;6(10):e1000948. doi: 10.1371/journal.pcbi.1000948 (PMC2951343; doi:10.1371/journal.pcbi.1000948)
Supplement: Table S1 — Consensus genotypes for different mutation rates and population structures. Mean probabilities for each gene averaged over 80 average LODs, with variance in brackets. SS: spatially-structured population, WM: well-mixed population, COOP: cooperator, DEFEC: defector. (0.04 MB DOC) [file pcbi.1000948.s005.doc]

|  | *P_C_* | *P_CC_* | *P_CD_* | *P_DC_* | *P_DD_* |
| --- | --- | --- | --- | --- | --- |
| SS COOP (*μ* =1%) | 0.647 (0.088) | 0.989 (0.005) | 0.234 (0.035) | 0.318 (0.075) | 0.448 (0.054) |
| SS DEFEC (*μ* =5%) | 0.481 (0.084) | 0.458 (0.091) | 0.31 5(0.062) | 0.243 (0.073) | 0.325 (0.064) |
| WM COOP (*μ* =1%) | 0.595 (0.098) | 0.893 (0.056) | 0.247 (0.038) | 0.247 (0.079) | 0.356 (0.076) |
| WM DEFEC (*μ* =5%) | 0.442 (0.081) | 0.460 (0.084) | 0.325 (0.059) | 0.063 (0.018) | 0.053 (0.012) |
