# Supplementary material for: Critical Dynamics in the Evolution of Stochastic Strategies for the Iterated Prisoner's Dilemma
Source: PLoS Comput Biol. 2010 Oct 7;6(10):e1000948. doi: 10.1371/journal.pcbi.1000948 (PMC2951343; doi:10.1371/journal.pcbi.1000948)
Supplement: Text S1 — Description of consensus strategies. (0.03 MB DOC) [file pcbi.1000948.s006.doc]

**Text S1. Description of consensus strategies**.

At low mutation rates (<1%) and a fixed replacement rate of 1%, robust cooperation quickly emerges as the dominant strategy for both well-mixed and spatially-structured populations. Robustness is generally regarded as a measure of a system’s performance in the face of perturbation or uncertainty. In this context, robust cooperation describes three basic behaviors that allow players to play well against defective players and even when faced with their own strategy, described in the main text.

The consensus genotypes at high mutation rates (5%) and a fixed replacement rate of 1%, for both population types, are representative of robust defection. Robustness in this case is less of a mandate in a cooperative regime, since cooperation is inherently a more risky behavior in PD. Robust defectors have to maintain D exchanges while at the same time be willing to bait cooperative strategists with C plays and exploit them in the process. A *P_CC_* probability close to 0.5 with a bias towards defection compared to the *P_CC_* of RC is indicative of the unwillingness to engage in extended cooperative exchanges but at the same time a willingness to establish limited CC exchanges in hopes of future exploitation of a cooperative strategist. On the other hand, a very low *P_DD_* probability expresses eagerness to maintain defective play. For the well-mixed population, the *P_DD_* probability is much smaller than the equivalent probability in the spatially-structured population, which might be due to the absence of clusters under well-mixing giving rise to a higher degree of uncertainty experienced by players. The same reasoning might be applicable to the low *P_DC_* probability. Defector strategists in well-mixed populations are more willing to take advantage of cooperative players since they play against many more such players due to the absence of cooperative cluster shielding.
