# Supplementary material for: Critical Dynamics in the Evolution of Stochastic Strategies for the Iterated Prisoner's Dilemma
Source: PLoS Comput Biol. 2010 Oct 7;6(10):e1000948. doi: 10.1371/journal.pcbi.1000948 (PMC2951343; doi:10.1371/journal.pcbi.1000948)
Supplement: Text S2 — Experimental statistics. (0.03 MB DOC) [file pcbi.1000948.s007.doc]

**Text S2. Experimental statistics**

For the spatially-structured phase transition experiments a total of 532 pairs of different mutation and replacement rate experiments were run, for a minimum of 13 and a maximum of 80 replicates for each pair (depending on the replacement rate), leading to 14,576 replicates in total. For the well-mixed phase transition experiments a total of 304 pairs of different mutation and replacement rate experiments were run, for a minimum of 10 and a maximum of 80 replicates each, leading to 10,080 replicates in total. For both population types, each experiment was run for 500,000 updates leading to approximately 7.3 billion updates for spatially-structured and 5 billion updates for well-mixed populations. All experiments were run in parallel on up to 25 dual core 2.6GHz (Intel® Pentium® Processor E5300) computers for an approximate total of about 8,500 hours for the spatially-structured and 5,900 hours for well-mixed population experiments. Data collected from the phase transition experiments were used to generate Figures 1, 2 and 3 as well as figures S1, S2, S3 and S4. For Figure 4A and memory depth 2, 16 different mutation rates were run at 1% replacement rate (80 replicates each), for a total of 1,280 experiments requiring 1,280 CPU hours. For Figure 4B, bits 1 to 5 were run for 40 replicate experiments at 15 different mutation rates (and a fixed replacement rate of 1%), for a total of 3,000 experiments (1,000 CPU hours). In total, the study required almost 29,000 individual experiments requiring a total of 1.9 CPU years (about 10 CPU weeks in parallel).
